# Supplementary material for: Altered microRNA Transcriptome in Cultured Human Liver Cells upon Infection with Ebola Virus
Source: Int J Mol Sci. 2021 Apr 6;22(7):3792. doi: 10.3390/ijms22073792 (PMC8038836; doi:10.3390/ijms22073792)
Supplement: Supplementary file 1 [file ijms-22-03792-s001.zip › Supplementary_File/C_ GO_Analysis_Results/16-30nt_go_Mayinga-96h-Huh7_vs_Control-96h-Huh7_up.mature_mirna_targets/CC_result(Human).html]

| GO.ID | Term | Ontology | Count | Pop.Hits | List.Total | Pop.Total | Fold.Enrichment | Pvalue | FDR | Enrichment.Score | Gene.Ratio | GENES |
| --- | --- | --- | --- | --- | --- | --- | --- | --- | --- | --- | --- | --- |
| GO:0005622 | intracellular | Cellular component | 359 | 14673 | 414 | 18698 | 1.10502052638008 | 9.47404478377924e-06 | 0.00813820446926637 | 5.02346456669459 | 0.867149758454106 | SAP30L//KAT7//NECAB3//DHCR24//COPG1//CUX2//ZDHHC17//HEPACAM2//HLA-DPB1//LMAN1//TMED7//B2M//CAMK1G//GALNT14//RAB36//EXOC8//ASB15//FBXW11//DCUN1D5//GTPBP1//RPL7L1//COA1//MAP9//RGS7//CKS2//CCNC//PSMA1//PSMC6//TRIAP1//WDHD1//DYNC1LI2//FBXO28//RASSF2//SPC24//ATRX//APEX1//H2AFY2//KLF2//TRPS1//MIXL1//NCOA1//HAND2//DDX6//UBE2I//C14ORF39//SYCE1//PCGF3//TCTEX1D2//CDC14A//TRIM71//EIF4E//ZC3H12A//MEX3B//E2F5//APEX2//YBX2//CDK12//DYNLT1//KRT6C//WDR1//MCCC2//SNRPF//CCSAP//DNALI1//CPQ//IFI30//TMEM199//RNF152//WDR48//LAPTM5//CTSF//RIPK3//COX4I1//CPOX//CRAT//DHFR//ALDH1B1//KIF1B//PPP1R13B//TIMM9//ACAD8//FAS//OXCT1//PCK1//MARC2//RFK//AGPAT5//BAK1//HIF3A//VDAC1//YWHAG//MUL1//NCOA4//SFXN1//EXOG//NAT2//ARPC5//NOD1//OSBPL8//CLTA//KLHDC1//MAP3K8//PSMA8//DGKA//EIF1AX//EIF5//FKBP1A//DZIP1//ALDOB//NCBP2//LARP4B//OTUD3//CRTC1//PIP5K1C//STEAP2//GJB2//GCLM//GSDMA//SATL1//INPP5B//KPNA1//AFF3//SULT6B1//SMAD5//MTM1//MYL1//NF2//NPM1//CRLF3//PDE1B//PEX5L//CMPK1//ATP7A//COMMD8//ETNK2//OGFOD1//PRKAA2//N4BP2//NGLY1//NUFIP2//PTPN7//RAP2C//ABCD3//ENOPH1//RPE//NECAB1//COPS7B//UPF3A//SP4//STX1A//BTF3//PRDX2//TCHH//TSN//VSNL1//XPNPEP1//YES1//CSDE1//ARHGAP28//PTP4A2//DUSP16//CDK2AP1//CLTCL1//PIP5K1A//RAB6C//STK24//CUL2//KRTAP4-4//C1ORF198//RGS5//DDX3Y//EIF3A//SYNJ1//IDI2//MOB1B//FAM114A1//CEP135//G3BP2//LACC1//MRPS7//RBM7//FSTL3//SPEG//USP16//ZNF273//SUB1//BTG3//ZHX1//FOXP4//ZNF488//ZNF597//ZNF558//IRX2//ZNF320//DR1//FGF14//EBF3//GABPB1//ALX3//SENP3//NR6A1//GFI1//TAF5L//ZNF544//HOXA9//TUBB2B//ZNF699//MCAM//MITF//NFYC//NOVA2//PAX7//PBX3//NMD3//DBR1//TAOK3//UFM1//PLAGL1//SNTG1//ACPP//UBE2W//MCM10//RNPC3//ARNTL2//REXO4//PURB//WIZ//RAD52//RARB//PBOV1//RFX4//RNF4//ALX4//RORA//ZNF704//SERPINB3//C11ORF1//AEN//SLC30A5//TACC1//XBP1//ZNF37A//ZNF75D//PGBD5//ZNF329//SLTM//PYROXD1//C2ORF16//CDKL1//FUBP3//PIAS2//ZNF251//ZNF804A//SRSF11//TEX30//QKI//CREB5//TRAF4//MFAP3L//CEP350//TMEM170A//BCHE//MTRNR2L9//CLEC4M//PCMTD1//CCR5//LIX1L//ASZ1//RUNDC3B//SAMD9L//HEPACAM//MAPKBP1//LRRC8B//NBPF14//CD209//PLCXD3//QRICH1//SERPINB4//HEPN1//TBCA//TNS1//TRPC5//TUFT1//ZNF185//SLC30A2//C3ORF20//NBPF3//CD1D//POP4//KDM2A//MFSD8//CTDSPL2//FAM222B//TAF11//USP51//RCN2//SCD//SLC25A35//SNX30//TFRC//F2R//LRAT//CYP19A1//CERS6//SUMF2//C2ORF66//ASPH//TMEM100//RPE65//THBS1//CPED1//EBPL//SEC11A//LTBP1//FAM20C//VWA1//COL23A1//H6PD//NUS1//CANT1//MOGAT3//AGPAT4//SMO//LZTR1//KERA//GZMK//NPPC//C5AR1//CCDC68//IGSF22//SPAG17//CTTNBP2//SYP//ATP6V0E1//CD3G//SNAP47//COX18//CHIC1//GRIK2//VNN1//TIMP2//EXD1//KRTAP4-12//FAM168B//KCNS1//EMC4//MOB3A//RUNDC1//EREG//ARL5B//IL2//GFRAL//ARL15//PGLYRP4//PIRT//CALCR//FSD1L//PYROXD2//CD40LG// |
| GO:0044464 | cell part | Cellular component | 396 | 16886 | 414 | 18698 | 1.05916401031984 | 3.31213948460137e-05 | 0.0142256390863629 | 4.47989138199672 | 0.956521739130435 | TSPAN5//BTN2A2//RIPK3//BTN2A1//SNAP47//CLTA//CCR5//CANT1//FAM168B//EXOC8//NKAIN2//DGKA//EIF5//F2R//F2RL2//PPP1R13B//CRTC1//LRRC8B//CADM2//SLC41A1//SLC24A2//STEAP2//GJB2//GSDMA//OR2L13//SLC9C2//SIRPB2//GRIK2//CD274//CD209//HLA-DPB1//HTR1F//FAS//INPP5B//KCNC4//KCNQ3//KCNS1//GFRAL//LTA//MCAM//CD200//ASPH//MTM1//NF2//TAOK3//CRLF3//CHIC1//PMP22//ATP7A//DLL4//ACPP//TMEM100//B2M//CAMK1G//RAP2C//RGS7//RPE65//HIF3A//CDH24//PIRT//SLC9A2//SMO//ST14//STX1A//TFRC//TRPC3//TRPC5//C5AR1//VDAC1//XPNPEP2//YES1//SLC30A2//CSDE1//CALCR//PTP4A2//PRRT1//SYT16//PIP5K1A//RGS5//NCOA1//ADAM23//VNN1//CD1D//COL23A1//CD3G//ZNF804A//CACNA2D4//CD40LG//TRAF4//RASSF2//MFAP3L//WDR1//MOB3A//RUNDC1//EREG//ARL5B//IL2//SMAD5//ARL15//PGLYRP4//SERPINB4//TUFT1//CCDC68//FSD1L//RAB6C//PYROXD2//CLTCL1//HYAL4//LYZL6//THBS1//TIMP2//TNS1//TMEM199//RAB36//HEPACAM2//WIZ//TACC1//CEP350//YWHAG//TCHH//ARPC5//PDE1B//DYNLT1//MUL1//ASB15//FBXW11//DCUN1D5//CKS2//CCNC//PSMA1//PSMC6//MTRNR2L9//CLEC4M//NOD1//CPQ//USP16//BTG3//WDHD1//PCMTD1//LIX1L//MAP3K8//ASZ1//CPOX//PSMA8//RUNDC3B//DDX6//DHCR24//E2F5//EIF4E//SAMD9L//HEPACAM//FKBP1A//DZIP1//NCBP2//MAPKBP1//OTUD3//NBPF14//SENP3//HOXA9//APEX1//PLCXD3//TUBB2B//KPNA1//MITF//NPM1//PCK1//NMD3//YBX2//UFM1//CMPK1//SNTG1//QRICH1//OGFOD1//UBE2W//RFK//PRKAA2//NGLY1//ARNTL2//NUFIP2//PTPN7//RARB//PBOV1//RNF4//SERPINB3//NECAB3//HEPN1//NECAB1//UPF3A//TBCA//PRDX2//TSN//UBE2I//XBP1//XPNPEP1//ZNF185//DNALI1//ARHGAP28//MAP9//ZC3H12A//DUSP16//C3ORF20//NBPF3//STK24//CDC14A//EIF3A//CDKL1//FUBP3//IDI2//MOB1B//QKI//G3BP2//SUB1//NR6A1//PBX3//ALX4//TRPS1//HAND2//PIP5K1C//PRSS22//SYP//CTTNBP2//SHISA6//SHISA9//KIF1B//ZDHHC17//FSTL3//SYNJ1//CUL2//GTPBP1//CDK12//NFYC//RORA//CCSAP//COX4I1//CRAT//TIMM9//SLC25A35//MRPS7//MARC2//ABCD3//SFXN1//EXOG//TSPAN19//TSPAN12//PTPRG//SLC30A5//LAPTM5//KAT7//TMEM170A//AGPAT5//BAK1//SNRPF//H2AFY2//DYNC1LI2//FBXO28//DHFR//ALDH1B1//ACAD8//APEX2//OXCT1//TRIAP1//COA1//MCCC2//NCOA4//RBM7//SPEG//PCGF3//ZNF273//ZHX1//FOXP4//ZNF488//ZNF597//SPC24//ZNF558//IRX2//ZNF320//DR1//FGF14//CUX2//EBF3//GABPB1//ALX3//GFI1//TAF5L//ZNF544//ZNF699//AFF3//NOVA2//PAX7//DBR1//PLAGL1//ATRX//MCM10//RNPC3//REXO4//WDR48//PURB//ENOPH1//RAD52//RFX4//ZNF704//C11ORF1//AEN//SP4//BTF3//ZNF37A//ZNF75D//PGBD5//ZNF329//SAP30L//SLTM//PYROXD1//CDK2AP1//MIXL1//C2ORF16//DDX3Y//PIAS2//ZNF251//FAM114A1//SRSF11//TEX30//CREB5//USP51//LARP4B//RPL7L1//RCN2//SCD//CYP19A1//COPG1//CERS6//SUMF2//C2ORF66//TMED7//CPED1//EBPL//IFI30//MFSD8//LMAN1//FAM20C//TAF11//GALNT14//LZTR1//CEP135//TCTEX1D2//NAT2//OSBPL8//KLHDC1//EIF1AX//ALDOB//GCLM//KRT6C//SATL1//SULT6B1//MYL1//PEX5L//COMMD8//ETNK2//N4BP2//RPE//COPS7B//VSNL1//MEX3B//KRTAP4-4//C1ORF198//LTBP1//BCHE//VWA1//H6PD//KERA//LRAT//TRIM71//LACC1//SNX30//SEC11A//NUS1//MOGAT3//AGPAT4//IGSF22//EXD1//EMC4//SPAG17//POP4//KDM2A//CTDSPL2//FAM222B//IGSF5//CTSF//RNF152//KLF2//ATP6V0E1//GZMK//NPPC//C14ORF39//SYCE1//COX18//KRTAP4-12// |
| GO:0005623 | cell | Cellular component | 396 | 16915 | 414 | 18698 | 1.05734812168258 | 4.97754856235688e-05 | 0.0142523807168819 | 4.30298449418137 | 0.956521739130435 | TSPAN5//BTN2A2//RIPK3//BTN2A1//SNAP47//CLTA//CCR5//CANT1//FAM168B//EXOC8//NKAIN2//DGKA//EIF5//F2R//F2RL2//PPP1R13B//CRTC1//LRRC8B//CADM2//SLC41A1//SLC24A2//STEAP2//GJB2//GSDMA//OR2L13//SLC9C2//SIRPB2//GRIK2//CD274//CD209//HLA-DPB1//HTR1F//FAS//INPP5B//KCNC4//KCNQ3//KCNS1//GFRAL//LTA//MCAM//CD200//ASPH//MTM1//NF2//TAOK3//CRLF3//CHIC1//PMP22//ATP7A//DLL4//ACPP//TMEM100//B2M//CAMK1G//RAP2C//RGS7//RPE65//HIF3A//CDH24//PIRT//SLC9A2//SMO//ST14//STX1A//TFRC//TRPC3//TRPC5//C5AR1//VDAC1//XPNPEP2//YES1//SLC30A2//CSDE1//CALCR//PTP4A2//PRRT1//SYT16//PIP5K1A//RGS5//NCOA1//ADAM23//VNN1//CD1D//COL23A1//CD3G//ZNF804A//CACNA2D4//CD40LG//TRAF4//RASSF2//MFAP3L//WDR1//SAP30L//KAT7//NECAB3//DHCR24//COPG1//CUX2//ZDHHC17//HEPACAM2//LMAN1//TMED7//GALNT14//RAB36//ASB15//FBXW11//DCUN1D5//GTPBP1//RPL7L1//COA1//MAP9//MOB3A//RUNDC1//EREG//ARL5B//IL2//SMAD5//ARL15//PGLYRP4//SERPINB4//TUFT1//CCDC68//FSD1L//RAB6C//PYROXD2//CKS2//CCNC//PSMA1//PSMC6//TRIAP1//WDHD1//DYNC1LI2//FBXO28//SPC24//ATRX//APEX1//H2AFY2//KLF2//TRPS1//MIXL1//HAND2//DDX6//UBE2I//C14ORF39//SYCE1//PCGF3//TCTEX1D2//CDC14A//TRIM71//EIF4E//ZC3H12A//MEX3B//TCHH//E2F5//APEX2//YBX2//CDK12//DYNLT1//KRT6C//PIP5K1C//MCCC2//SNRPF//CCSAP//DNALI1//CPQ//IFI30//TMEM199//RNF152//WDR48//LAPTM5//CTSF//COX4I1//CPOX//CRAT//DHFR//ALDH1B1//KIF1B//TIMM9//ACAD8//OXCT1//PCK1//MARC2//RFK//AGPAT5//BAK1//YWHAG//MUL1//NCOA4//SFXN1//EXOG//NAT2//ARPC5//NOD1//OSBPL8//KLHDC1//MAP3K8//PSMA8//EIF1AX//FKBP1A//DZIP1//ALDOB//NCBP2//LARP4B//OTUD3//GCLM//SATL1//KPNA1//AFF3//SULT6B1//MYL1//NPM1//PDE1B//PEX5L//CMPK1//COMMD8//ETNK2//OGFOD1//PRKAA2//N4BP2//NGLY1//NUFIP2//PTPN7//ABCD3//ENOPH1//RPE//NECAB1//COPS7B//UPF3A//SP4//BTF3//PRDX2//TSN//VSNL1//XPNPEP1//ARHGAP28//DUSP16//CDK2AP1//CLTCL1//STK24//CUL2//KRTAP4-4//C1ORF198//DDX3Y//EIF3A//SYNJ1//IDI2//MOB1B//FAM114A1//CEP135//G3BP2//LACC1//PRSS22//CLEC4M//TSPAN19//TSPAN12//PTPRG//SLC30A5//MRPS7//RBM7//FSTL3//SPEG//USP16//ZNF273//SUB1//BTG3//ZHX1//FOXP4//ZNF488//ZNF597//ZNF558//IRX2//ZNF320//DR1//FGF14//EBF3//GABPB1//ALX3//SENP3//NR6A1//GFI1//TAF5L//ZNF544//HOXA9//TUBB2B//ZNF699//MITF//NFYC//NOVA2//PAX7//PBX3//NMD3//DBR1//UFM1//PLAGL1//SNTG1//UBE2W//MCM10//RNPC3//ARNTL2//REXO4//PURB//WIZ//RAD52//RARB//PBOV1//RFX4//RNF4//ALX4//RORA//ZNF704//SERPINB3//C11ORF1//AEN//TACC1//XBP1//ZNF37A//ZNF75D//PGBD5//ZNF329//SLTM//PYROXD1//C2ORF16//CDKL1//FUBP3//PIAS2//ZNF251//SRSF11//TEX30//QKI//CREB5//CEP350//TMEM170A//BCHE//MTRNR2L9//PCMTD1//LIX1L//ASZ1//RUNDC3B//SAMD9L//HEPACAM//MAPKBP1//NBPF14//PLCXD3//QRICH1//HEPN1//TBCA//TNS1//ZNF185//C3ORF20//NBPF3//POP4//KDM2A//MFSD8//CTDSPL2//FAM222B//TAF11//USP51//RCN2//SCD//SLC25A35//SNX30//LRAT//CYP19A1//CERS6//SUMF2//C2ORF66//THBS1//CPED1//EBPL//SEC11A//LTBP1//FAM20C//VWA1//H6PD//NUS1//MOGAT3//AGPAT4//LZTR1//KERA//GZMK//NPPC//IGSF22//SPAG17//CTTNBP2//SYP//SHISA9//HYAL4//LYZL6//TIMP2//ATP6V0E1//SHISA6//IGSF5//COX18//EXD1//KRTAP4-12//EMC4// |
| GO:0036464 | cytoplasmic ribonucleoprotein granule | Cellular component | 13 | 191 | 414 | 18698 | 3.0740066267041 | 0.0003456504877657 | 0.0742284422476841 | 3.46136282592518 | 0.0314009661835749 | TRIM71//DDX6//EIF4E//ZC3H12A//MEX3B//LARP4B//OGFOD1//NUFIP2//EXD1//ASZ1//GABPB1//TAF5L//DDX3Y// |
| GO:0035770 | ribonucleoprotein granule | Cellular component | 13 | 200 | 414 | 18698 | 2.93567632850242 | 0.000536378775610646 | 0.077323991169317 | 3.27052841541691 | 0.0314009661835749 | DDX6//EIF4E//GABPB1//TAF5L//ZC3H12A//DDX3Y//TRIM71//MEX3B//LARP4B//OGFOD1//NUFIP2//EXD1//ASZ1// |
| GO:0044424 | intracellular part | Cellular component | 346 | 14395 | 414 | 18698 | 1.08557352677141 | 0.000540097726444589 | 0.077323991169317 | 3.26752765088886 | 0.835748792270531 | ASB15//FBXW11//DCUN1D5//RGS7//CKS2//CCNC//PSMA1//PSMC6//MTRNR2L9//ARPC5//CLEC4M//NOD1//CPQ//USP16//BTG3//WDHD1//PCMTD1//CCR5//LIX1L//MAP3K8//ASZ1//CPOX//PSMA8//RUNDC3B//DDX6//DHCR24//E2F5//EIF4E//EIF5//SAMD9L//HEPACAM//FKBP1A//DZIP1//NCBP2//MAPKBP1//OTUD3//PPP1R13B//CRTC1//LRRC8B//NBPF14//SENP3//GSDMA//CD209//HOXA9//APEX1//PLCXD3//TUBB2B//KPNA1//SMAD5//MITF//MTM1//NF2//NPM1//PCK1//NMD3//YBX2//TAOK3//CRLF3//UFM1//CMPK1//SNTG1//QRICH1//OGFOD1//UBE2W//RFK//PRKAA2//NGLY1//ARNTL2//NUFIP2//PTPN7//RAP2C//RARB//PBOV1//RNF4//SERPINB3//SERPINB4//NECAB3//HEPN1//NECAB1//UPF3A//TACC1//TBCA//PRDX2//TNS1//TRPC5//TSN//TUFT1//UBE2I//XBP1//XPNPEP1//ZNF185//SLC30A2//DNALI1//ARHGAP28//MAP9//ZC3H12A//PTP4A2//DUSP16//C3ORF20//NBPF3//STK24//RGS5//CDC14A//EIF3A//CDKL1//FUBP3//CD1D//IDI2//ZNF804A//MOB1B//QKI//TRAF4//RASSF2//MFAP3L//G3BP2//SUB1//NR6A1//PBX3//ALX4//TRPS1//HAND2//CAMK1G//YES1//CUL2//GTPBP1//CDK12//ZDHHC17//COX4I1//CRAT//TIMM9//SLC25A35//MRPS7//MARC2//ABCD3//VDAC1//CSDE1//SFXN1//EXOG//KAT7//AGPAT5//BAK1//SNRPF//IFI30//ALDH1B1//MFSD8//APEX2//PLAGL1//WDR48//SMO//CCSAP//TCTEX1D2//RIPK3//DHFR//KIF1B//ACAD8//FAS//OXCT1//TRIAP1//COA1//MCCC2//HIF3A//DYNLT1//YWHAG//MUL1//NCOA4//NAT2//OSBPL8//CLTA//KLHDC1//SPC24//EXOC8//DGKA//DYNC1LI2//EIF1AX//COPG1//ALDOB//LARP4B//PIP5K1C//STEAP2//GJB2//GCLM//KRT6C//SATL1//INPP5B//AFF3//SULT6B1//MYL1//PDE1B//PEX5L//ATP7A//COMMD8//ETNK2//N4BP2//B2M//ENOPH1//RPE//COPS7B//SP4//STX1A//BTF3//TCHH//VSNL1//CDK2AP1//CLTCL1//PIP5K1A//RAB6C//MEX3B//KRTAP4-4//C1ORF198//NCOA1//DDX3Y//SYNJ1//FAM114A1//CEP135//WDR1//TMEM199//CYP19A1//CERS6//SUMF2//C2ORF66//ASPH//TMED7//TMEM100//RCN2//RPE65//SCD//THBS1//CPED1//EBPL//LMAN1//FSTL3//F2R//HEPACAM2//FAM20C//SLC30A5//TAF11//GALNT14//LZTR1//RAB36//SNAP47//CHIC1//TFRC//GABPB1//TAF5L//FAM168B//KCNS1//LRAT//LTBP1//BCHE//VWA1//COL23A1//H6PD//KERA//CANT1//TIMP2//DR1//RNF152//LAPTM5//CTSF//CTTNBP2//NFYC//RORA//LACC1//RBM7//SPEG//PCGF3//ZNF273//ZHX1//FOXP4//ZNF488//ZNF597//ZNF558//IRX2//ZNF320//FGF14//CUX2//EBF3//ALX3//GFI1//ZNF544//ZNF699//MCAM//NOVA2//PAX7//DBR1//ATRX//ACPP//MCM10//RNPC3//REXO4//PURB//WIZ//RAD52//RFX4//ZNF704//C11ORF1//AEN//ZNF37A//ZNF75D//PGBD5//ZNF329//SAP30L//SLTM//PYROXD1//MIXL1//C2ORF16//PIAS2//ZNF251//SRSF11//TEX30//CREB5//CEP350//SNX30//GZMK//NPPC//TMEM170A//H2AFY2//TRIM71//EXD1//FBXO28//USP51//RPL7L1//CCDC68//KLF2//C14ORF39//SYCE1//POP4//KDM2A//CTDSPL2//FAM222B//IGSF22//SPAG17//HLA-DPB1//SEC11A//NUS1//MOGAT3//AGPAT4//EMC4//C5AR1//ATP6V0E1//SYP//KRTAP4-12//GRIK2//VNN1//CD3G//COX18// |
| GO:0030660 | Golgi-associated vesicle membrane | Cellular component | 9 | 113 | 414 | 18698 | 3.5971527510581 | 0.000920835346126741 | 0.11299965176041 | 3.03581801872972 | 0.0217391304347826 | HLA-DPB1//LMAN1//B2M//TMEM199//TMED7//CLTA//CLTCL1//COPG1//ZDHHC17// |
| GO:0005798 | Golgi-associated vesicle | Cellular component | 11 | 174 | 414 | 18698 | 2.85521128324727 | 0.00175855527474796 | 0.188824872626062 | 2.75484397642821 | 0.0265700483091787 | LMAN1//TMED7//STEAP2//ATP7A//ZDHHC17//TMEM199//HLA-DPB1//B2M//CLTA//CLTCL1//COPG1// |
| GO:0010494 | cytoplasmic stress granule | Cellular component | 5 | 45 | 414 | 18698 | 5.01825013419216 | 0.00306387157532803 | 0.292429520356309 | 2.51372944246478 | 0.0120772946859903 | DDX6//EIF4E//LARP4B//OGFOD1//NUFIP2// |
| GO:0005667 | transcription factor complex | Cellular component | 16 | 345 | 414 | 18698 | 2.09457396905412 | 0.00453217481744405 | 0.333103725101338 | 2.3436933466491 | 0.0386473429951691 | NFYC//RARB//RORA//TAF11//TAF5L//SUB1//E2F5//NR6A1//HOXA9//APEX1//SMAD5//PBX3//ARNTL2//ALX4//TRPS1//HAND2// |
| GO:0030662 | coated vesicle membrane | Cellular component | 10 | 171 | 414 | 18698 | 2.64118428115377 | 0.00487663506176952 | 0.333103725101338 | 2.31187974319659 | 0.0241545893719807 | HLA-DPB1//LMAN1//B2M//TMEM199//TFRC//CD3G//TMED7//CLTA//CLTCL1//COPG1// |
| GO:0030140 | trans-Golgi network transport vesicle | Cellular component | 4 | 32 | 414 | 18698 | 5.64553140096618 | 0.00521605294242861 | 0.333103725101338 | 2.28265800920713 | 0.00966183574879227 | CLTA//CLTCL1//STEAP2//ATP7A// |
| GO:0043186 | P granule | Cellular component | 3 | 17 | 414 | 18698 | 7.97016197783461 | 0.00581671231259612 | 0.333103725101338 | 2.23532241533893 | 0.0072463768115942 | ASZ1//EXD1//DDX6// |
| GO:0045495 | pole plasm | Cellular component | 3 | 17 | 414 | 18698 | 7.97016197783461 | 0.00581671231259612 | 0.333103725101338 | 2.23532241533893 | 0.0072463768115942 | EXD1//DDX6//ASZ1// |
| GO:0060293 | germ plasm | Cellular component | 3 | 17 | 414 | 18698 | 7.97016197783461 | 0.00581671231259612 | 0.333103725101338 | 2.23532241533893 | 0.0072463768115942 | EXD1//DDX6//ASZ1// |
| GO:0030663 | COPI-coated vesicle membrane | Cellular component | 3 | 18 | 414 | 18698 | 7.52737520128824 | 0.0068667430745976 | 0.364498216719342 | 2.16324920182262 | 0.0072463768115942 | COPG1//TMED7//TMEM199// |
| GO:0032991 | protein-containing complex | Cellular component | 135 | 5071 | 414 | 18698 | 1.20236125281867 | 0.00721358519700676 | 0.364498216719342 | 2.14184883454752 | 0.326086956521739 | DYNC1LI2//FBXO28//RASSF2//WDR1//COL23A1//THBS1//KPNA1//PURB//SUB1//E2F5//NR6A1//HOXA9//APEX1//SMAD5//PBX3//ARNTL2//ALX4//TRPS1//HAND2//PSMA8//PSMA1//EIF3A//KIF1B//ARPC5//CAMK1G//AFF3//COPS7B//EIF4E//PSMC6//CCNC//GFI1//SPC24//WDHD1//MCM10//PCGF3//NFYC//NPM1//RAD52//NCBP2//UPF3A//GFRAL//PEX5L//CSDE1//DZIP1//CCDC68//UBE2I//EXD1//PIP5K1A//DNALI1//CDK12//SNAP47//H2AFY2//BAK1//TRIAP1//SEC11A//VDAC1//SYNJ1//STX1A//CLTA//CLTCL1//EMC4//MCCC2//MRPS7//TIMM9//EXOC8//SNRPF//SAP30L//POP4//GCLM//MAPKBP1//TMEM199//LARP4B//NUFIP2//RARB//RORA//RNPC3//TCTEX1D2//DYNLT1//IGSF22//MYL1//COPG1//TMED7//DDX6//ATP6V0E1//TRIM71//ZC3H12A//MEX3B//OGFOD1//GABPB1//TAF5L//DDX3Y//CD3G//B2M//TFRC//RPL7L1//COA1//TAF11//COX4I1//RGS7//CACNA2D4//GJB2//KCNC4//KCNQ3//KCNS1//SNTG1//CDH24//FAS//LRRC8B//HLA-DPB1//FKBP1A//SHISA9//ASB15//FBXW11//DCUN1D5//ATRX//GTPBP1//CUL2//CKS2//RNF4//SHISA6//GRIK2//KAT7//DR1//TRPC5//SENP3//ASZ1//CALCR//PRKAA2//F2RL2//SLC41A1//LTBP1//MITF//NPPC//PGLYRP4//NCOA1// |
| GO:0043229 | intracellular organelle | Cellular component | 301 | 12604 | 414 | 18698 | 1.07858137206653 | 0.0107295047773155 | 0.499537154565958 | 1.96942032253009 | 0.727053140096618 | CPQ//IFI30//ALDH1B1//ZDHHC17//MFSD8//APEX2//PLAGL1//WDR48//ABCD3//UPF3A//SMO//SLC30A2//CDKL1//RIPK3//COX4I1//CPOX//CRAT//DDX6//DHFR//KIF1B//PPP1R13B//TIMM9//ACAD8//APEX1//FAS//OXCT1//PCK1//TRIAP1//MARC2//RFK//AGPAT5//COA1//BAK1//MCCC2//HIF3A//DYNLT1//VDAC1//YWHAG//MUL1//NCOA4//SFXN1//EXOG//RBM7//FSTL3//SPEG//PCGF3//USP16//ZNF273//SUB1//BTG3//KAT7//ZHX1//FOXP4//ZNF488//ASB15//PSMA8//ZNF597//SPC24//ZNF558//IRX2//ZNF320//DHCR24//DR1//E2F5//FGF14//DZIP1//NCBP2//FBXW11//CUX2//CRTC1//EBF3//GABPB1//ALX3//SENP3//NR6A1//GFI1//TAF5L//ZNF544//HOXA9//TUBB2B//ZNF699//KPNA1//AFF3//SMAD5//MCAM//MITF//NF2//NFYC//NOVA2//NPM1//PAX7//PBX3//NMD3//YBX2//DBR1//TAOK3//CRLF3//UFM1//CMPK1//CDK12//ATP7A//SNTG1//ATRX//ACPP//OGFOD1//UBE2W//MCM10//RNPC3//PRKAA2//NGLY1//PSMA1//ARNTL2//PSMC6//REXO4//NUFIP2//PURB//ENOPH1//WIZ//RAD52//RARB//PBOV1//RFX4//RNF4//ALX4//RORA//ZNF704//SERPINB3//NECAB3//NECAB1//C11ORF1//AEN//SLC30A5//SP4//TACC1//BTF3//TRPS1//TSN//UBE2I//XBP1//ZNF37A//ZNF75D//PGBD5//ZNF329//SAP30L//SLTM//PYROXD1//ZC3H12A//PTP4A2//DUSP16//CDK2AP1//MIXL1//PIP5K1A//RAB6C//C2ORF16//STK24//CDC14A//NCOA1//DDX3Y//EIF3A//CCNC//FUBP3//PIAS2//ZNF251//ZNF804A//MOB1B//FAM114A1//SRSF11//TEX30//QKI//CREB5//TRAF4//RASSF2//MFAP3L//CEP350//TMEM199//CYP19A1//COPG1//CERS6//SUMF2//C2ORF66//ASPH//TMED7//TMEM100//RCN2//RPE65//SCD//THBS1//CPED1//EBPL//CD1D//GJB2//INPP5B//LMAN1//F2R//HEPACAM2//B2M//FAM20C//TAF11//YES1//CSDE1//GALNT14//LZTR1//RAB36//DYNC1LI2//FBXO28//WDR1//MRPS7//USP51//LARP4B//RPL7L1//TBCA//CUL2//CCSAP//CCDC68//CEP135//CLTA//CLTCL1//PTPN7//TCHH//TNS1//CHIC1//TFRC//MYL1//RNF152//LAPTM5//CTSF//H2AFY2//LRAT//FKBP1A//LACC1//IDI2//MAP9//ARPC5//ZNF185//CCR5//STEAP2//SNX30//SAMD9L//EXOC8//MTM1//GZMK//NPPC//STX1A//TRIM71//EIF4E//MEX3B//EXD1//PEX5L//SYP//CTTNBP2//ASZ1//HLA-DPB1//KERA//C5AR1//TCTEX1D2//SNAP47//IGSF22//ALDOB//SPAG17//SYNJ1//CD3G//CANT1//TIMP2//VNN1//KRT6C//ATP6V0E1//RAP2C//MAPKBP1//CAMK1G//WDHD1//KLF2//HAND2//C14ORF39//SYCE1//SNRPF//DNALI1//TMEM170A//BCHE//POP4//KDM2A//PIP5K1C//CTDSPL2//QRICH1//COMMD8//FAM222B//COPS7B//SLC25A35//SEC11A//LTBP1//VWA1//COL23A1//H6PD//OSBPL8//NUS1//LRRC8B//MOGAT3//AGPAT4//COX18//KRTAP4-12//KRTAP4-4//EMC4// |
| GO:0031981 | nuclear lumen | Cellular component | 110 | 4071 | 414 | 18698 | 1.22035559637687 | 0.0110491338029723 | 0.499537154565958 | 1.95667176716751 | 0.265700483091787 | RBM7//FSTL3//PCGF3//USP16//POP4//KAT7//WDHD1//ZHX1//E2F5//ALDH1B1//DZIP1//NCBP2//KDM2A//PPP1R13B//CRTC1//PIP5K1C//GABPB1//MFSD8//SENP3//NR6A1//APEX1//KPNA1//AFF3//SMAD5//MITF//NFYC//NPM1//OXCT1//NMD3//DBR1//CTDSPL2//TRIAP1//CDK12//PLAGL1//ATRX//QRICH1//COMMD8//UBE2W//MCM10//H2AFY2//RNPC3//PRKAA2//FAM222B//PSMA1//PSMC6//NUFIP2//WDR48//PTPN7//WIZ//RAD52//RARB//RNF4//RORA//NECAB1//HIF3A//COPS7B//C11ORF1//AEN//SNRPF//SP4//TAF11//TRPS1//UBE2I//XBP1//SAP30L//SLTM//ZC3H12A//DUSP16//CDK2AP1//PIP5K1A//MEX3B//STK24//CUL2//CDC14A//NCOA1//CDKL1//CCNC//PIAS2//FAM114A1//SRSF11//RASSF2//SUB1//SPC24//DDX6//LARP4B//RPL7L1//NF2//CMPK1//ARNTL2//REXO4//RCN2//SCD//SLC30A5//UPF3A//TBCA//DDX3Y//EIF3A//MOB1B//GFI1//KLF2//MIXL1//HAND2//C14ORF39//SYCE1//PURB//DR1//FAS//TAF5L//APEX2//YBX2// |
| GO:0005868 | cytoplasmic dynein complex | Cellular component | 3 | 22 | 414 | 18698 | 6.15876152832675 | 0.0121403793112118 | 0.508420109039109 | 1.91576774405213 | 0.0072463768115942 | DYNC1LI2//TCTEX1D2//DYNLT1// |
| GO:0043226 | organelle | Cellular component | 321 | 13597 | 414 | 18698 | 1.06624436549836 | 0.0137990768853763 | 0.508420109039109 | 1.86014996555503 | 0.77536231884058 | CCSAP//SMO//SPAG17//CPQ//IFI30//ALDH1B1//ZDHHC17//MFSD8//APEX2//PLAGL1//WDR48//ABCD3//UPF3A//SLC30A2//CDKL1//SERPINB3//EDIL3//TFRC//CTSF//GRIK2//SHISA6//STX1A//SHISA9//RIPK3//COX4I1//CPOX//CRAT//DDX6//DHFR//KIF1B//PPP1R13B//TIMM9//ACAD8//APEX1//FAS//OXCT1//PCK1//TRIAP1//MARC2//RFK//AGPAT5//COA1//BAK1//MCCC2//HIF3A//DYNLT1//VDAC1//YWHAG//MUL1//NCOA4//SFXN1//EXOG//LACC1//IDI2//RBM7//FSTL3//SPEG//PCGF3//USP16//ZNF273//SUB1//BTG3//KAT7//ZHX1//FOXP4//ZNF488//ASB15//PSMA8//ZNF597//SPC24//ZNF558//IRX2//ZNF320//DHCR24//DR1//E2F5//FGF14//DZIP1//NCBP2//FBXW11//CUX2//CRTC1//EBF3//GABPB1//ALX3//SENP3//NR6A1//GFI1//TAF5L//ZNF544//HOXA9//TUBB2B//ZNF699//KPNA1//AFF3//SMAD5//MCAM//MITF//NF2//NFYC//NOVA2//NPM1//PAX7//PBX3//NMD3//YBX2//DBR1//TAOK3//CRLF3//UFM1//CMPK1//CDK12//ATP7A//SNTG1//ATRX//ACPP//OGFOD1//UBE2W//MCM10//RNPC3//PRKAA2//NGLY1//PSMA1//ARNTL2//PSMC6//REXO4//NUFIP2//PURB//ENOPH1//WIZ//RAD52//RARB//PBOV1//RFX4//RNF4//ALX4//RORA//ZNF704//NECAB3//NECAB1//C11ORF1//AEN//SLC30A5//SP4//TACC1//BTF3//TRPS1//TSN//UBE2I//XBP1//ZNF37A//ZNF75D//PGBD5//ZNF329//SAP30L//SLTM//PYROXD1//ZC3H12A//PTP4A2//DUSP16//CDK2AP1//MIXL1//PIP5K1A//RAB6C//C2ORF16//STK24//CDC14A//NCOA1//DDX3Y//EIF3A//CCNC//FUBP3//PIAS2//ZNF251//ZNF804A//MOB1B//FAM114A1//SRSF11//TEX30//QKI//CREB5//TRAF4//RASSF2//MFAP3L//CEP350//TMEM199//CYP19A1//COPG1//CERS6//SUMF2//C2ORF66//ASPH//TMED7//TMEM100//RCN2//RPE65//SCD//THBS1//CPED1//EBPL//CD1D//LRAT//GJB2//INPP5B//LMAN1//F2R//HEPACAM2//B2M//FAM20C//TAF11//YES1//CSDE1//GALNT14//LZTR1//RAB36//FKBP1A//CLTA//CHIC1//ARPC5//BTN2A2//SEMA3C//BTN2A1//CANT1//FAM168B//EIF4E//ALDOB//SEC11A//KRT6C//GPX3//CD274//CD209//MTM1//ARL15//H2AFY2//RAP2C//PTPRG//RPE//SERPINB4//VWA1//ST14//TBCA//PRDX2//TIMP2//XPNPEP1//XPNPEP2//CLTCL1//VNN1//WDR1//DYNC1LI2//FBXO28//MRPS7//USP51//LARP4B//RPL7L1//CUL2//CCDC68//CEP135//PTPN7//TCHH//TNS1//ZNF185//MYL1//MAP9//CCR5//STEAP2//SNX30//RNF152//LAPTM5//SAMD9L//EXOC8//PEX5L//SYP//CTTNBP2//GZMK//NPPC//TRIM71//MEX3B//EXD1//ASZ1//HLA-DPB1//KERA//C5AR1//TCTEX1D2//SNAP47//IGSF22//SYNJ1//CD3G//LYZL6//ATP6V0E1//MAPKBP1//CAMK1G//WDHD1//KLF2//HAND2//C14ORF39//SYCE1//SNRPF//DNALI1//TMEM170A//BCHE//POP4//KDM2A//PIP5K1C//CTDSPL2//QRICH1//COMMD8//FAM222B//COPS7B//SLC25A35//LTBP1//COL23A1//H6PD//OSBPL8//NUS1//LRRC8B//MOGAT3//AGPAT4//COX18//KRTAP4-12//KRTAP4-4//EMC4// |
| GO:0005737 | cytoplasm | Cellular component | 277 | 11544 | 414 | 18698 | 1.08372293698381 | 0.0156698319523894 | 0.508420109039109 | 1.80493566100054 | 0.669082125603865 | CTTNBP2//CLTA//GRIK2//KIF1B//FKBP1A//THBS1//EXD1//DDX6//CCSAP//TCTEX1D2//DNALI1//NECAB3//DHCR24//COPG1//CUX2//ZDHHC17//HEPACAM2//HLA-DPB1//LMAN1//TMED7//B2M//CAMK1G//GALNT14//RAB36//EXOC8//GTPBP1//RPL7L1//COA1//MAP9//TRIAP1//TRIM71//EIF4E//ZC3H12A//MEX3B//MCCC2//CPQ//IFI30//TMEM199//DYNC1LI2//RNF152//WDR48//LAPTM5//CTSF//RIPK3//COX4I1//CPOX//CRAT//DHFR//ALDH1B1//PPP1R13B//TIMM9//ACAD8//APEX2//APEX1//FAS//OXCT1//PCK1//MARC2//RFK//AGPAT5//BAK1//HIF3A//DYNLT1//VDAC1//YWHAG//MUL1//NCOA4//SFXN1//EXOG//NAT2//ARPC5//NOD1//KAT7//OSBPL8//KLHDC1//MAP3K8//ASB15//PSMA8//SPC24//DGKA//EIF1AX//EIF5//DZIP1//ALDOB//NCBP2//LARP4B//OTUD3//FBXW11//CRTC1//PIP5K1C//STEAP2//GJB2//GCLM//GSDMA//KRT6C//SATL1//INPP5B//KPNA1//AFF3//SULT6B1//SMAD5//MTM1//MYL1//NF2//NPM1//CRLF3//PDE1B//PEX5L//CMPK1//ATP7A//COMMD8//ETNK2//OGFOD1//PRKAA2//N4BP2//NGLY1//PSMA1//PSMC6//NUFIP2//PTPN7//RAP2C//ABCD3//ENOPH1//RGS7//RPE//NECAB1//COPS7B//UPF3A//SNRPF//SP4//STX1A//BTF3//PRDX2//TCHH//TSN//UBE2I//VSNL1//XPNPEP1//YES1//CSDE1//ARHGAP28//PTP4A2//DUSP16//CDK2AP1//CLTCL1//PIP5K1A//RAB6C//STK24//CUL2//KRTAP4-4//C1ORF198//RGS5//CDC14A//NCOA1//DDX3Y//EIF3A//SYNJ1//IDI2//MOB1B//FAM114A1//CEP135//RASSF2//G3BP2//WDR1//LACC1//MRPS7//SLC25A35//MFSD8//ACPP//SLC30A2//CD1D//CCR5//SNX30//TFRC//F2R//SAMD9L//LRAT//CYP19A1//CERS6//SUMF2//C2ORF66//ASPH//UFM1//TMEM100//RCN2//RPE65//SCD//XBP1//CPED1//EBPL//SEC11A//FSTL3//LTBP1//FAM20C//BCHE//VWA1//COL23A1//H6PD//NUS1//TMEM170A//CANT1//LRRC8B//MOGAT3//AGPAT4//PLAGL1//SLC30A5//SMO//TAF11//LZTR1//KERA//GZMK//NPPC//C5AR1//CCDC68//CEP350//IGSF22//SYP//ATP6V0E1//PYROXD1//CD3G//SNAP47//COX18//CHIC1//SERPINB3//VNN1//TIMP2//GABPB1//TAF5L//FAM168B//KCNS1//TRAF4//ASZ1//EMC4//SPAG17//MTRNR2L9//CLEC4M//USP16//BTG3//WDHD1//PCMTD1//LIX1L//RUNDC3B//E2F5//HEPACAM//MAPKBP1//NBPF14//SENP3//CD209//HOXA9//PLCXD3//TUBB2B//MITF//NMD3//YBX2//TAOK3//SNTG1//QRICH1//UBE2W//ARNTL2//RARB//PBOV1//RNF4//SERPINB4//HEPN1//TACC1//TBCA//TNS1//TRPC5//TUFT1//ZNF185//C3ORF20//NBPF3//CDKL1//FUBP3//ZNF804A//QKI//MFAP3L// |
| GO:0031974 | membrane-enclosed lumen | Cellular component | 135 | 5188 | 414 | 18698 | 1.17524554993128 | 0.0156853310022845 | 0.508420109039109 | 1.8045063120986 | 0.326086956521739 | ARPC5//DYNLT1//ALDH1B1//ACAD8//OXCT1//MCCC2//FSTL3//SUMF2//LTBP1//B2M//FAM20C//BCHE//RCN2//VWA1//THBS1//COL23A1//H6PD//KERA//CANT1//TIMP2//CPOX//TIMM9//TRIAP1//CRAT//ABCD3//SERPINB3//IFI30//CTSF//VDAC1//RBM7//PCGF3//USP16//POP4//KAT7//WDHD1//ZHX1//E2F5//DZIP1//NCBP2//KDM2A//PPP1R13B//CRTC1//PIP5K1C//GABPB1//MFSD8//SENP3//NR6A1//APEX1//KPNA1//AFF3//SMAD5//MITF//NFYC//NPM1//NMD3//DBR1//CTDSPL2//CDK12//PLAGL1//ATRX//QRICH1//COMMD8//UBE2W//MCM10//H2AFY2//RNPC3//PRKAA2//FAM222B//PSMA1//PSMC6//NUFIP2//WDR48//PTPN7//WIZ//RAD52//RARB//RNF4//RORA//NECAB1//HIF3A//COPS7B//C11ORF1//AEN//SNRPF//SP4//TAF11//TRPS1//UBE2I//XBP1//SAP30L//SLTM//ZC3H12A//DUSP16//CDK2AP1//PIP5K1A//MEX3B//STK24//CUL2//CDC14A//NCOA1//CDKL1//CCNC//PIAS2//FAM114A1//SRSF11//RASSF2//SUB1//SPC24//DDX6//LARP4B//RPL7L1//NF2//CMPK1//ARNTL2//REXO4//SCD//SLC30A5//UPF3A//TBCA//DDX3Y//EIF3A//MOB1B//MRPS7//GFI1//KLF2//MIXL1//HAND2//C14ORF39//SYCE1//PURB//DR1//FAS//TAF5L//APEX2//YBX2// |
| GO:0043233 | organelle lumen | Cellular component | 135 | 5188 | 414 | 18698 | 1.17524554993128 | 0.0156853310022845 | 0.508420109039109 | 1.8045063120986 | 0.326086956521739 | ALDH1B1//ACAD8//OXCT1//MCCC2//FSTL3//SUMF2//LTBP1//B2M//FAM20C//BCHE//RCN2//VWA1//THBS1//COL23A1//H6PD//KERA//CANT1//TIMP2//ARPC5//DYNLT1//SERPINB3//IFI30//CTSF//CRAT//ABCD3//CPOX//TIMM9//TRIAP1//VDAC1//RBM7//PCGF3//USP16//POP4//KAT7//WDHD1//ZHX1//E2F5//DZIP1//NCBP2//KDM2A//PPP1R13B//CRTC1//PIP5K1C//GABPB1//MFSD8//SENP3//NR6A1//APEX1//KPNA1//AFF3//SMAD5//MITF//NFYC//NPM1//NMD3//DBR1//CTDSPL2//CDK12//PLAGL1//ATRX//QRICH1//COMMD8//UBE2W//MCM10//H2AFY2//RNPC3//PRKAA2//FAM222B//PSMA1//PSMC6//NUFIP2//WDR48//PTPN7//WIZ//RAD52//RARB//RNF4//RORA//NECAB1//HIF3A//COPS7B//C11ORF1//AEN//SNRPF//SP4//TAF11//TRPS1//UBE2I//XBP1//SAP30L//SLTM//ZC3H12A//DUSP16//CDK2AP1//PIP5K1A//MEX3B//STK24//CUL2//CDC14A//NCOA1//CDKL1//CCNC//PIAS2//FAM114A1//SRSF11//RASSF2//SUB1//SPC24//DDX6//LARP4B//RPL7L1//NF2//CMPK1//ARNTL2//REXO4//SCD//SLC30A5//UPF3A//TBCA//DDX3Y//EIF3A//MOB1B//MRPS7//GFI1//KLF2//MIXL1//HAND2//C14ORF39//SYCE1//PURB//DR1//FAS//TAF5L//APEX2//YBX2// |
| GO:0070013 | intracellular organelle lumen | Cellular component | 135 | 5188 | 414 | 18698 | 1.17524554993128 | 0.0156853310022845 | 0.508420109039109 | 1.8045063120986 | 0.326086956521739 | ALDH1B1//ACAD8//OXCT1//MCCC2//FSTL3//SUMF2//LTBP1//B2M//FAM20C//BCHE//RCN2//VWA1//THBS1//COL23A1//H6PD//KERA//CANT1//TIMP2//ARPC5//DYNLT1//SERPINB3//IFI30//CTSF//CRAT//ABCD3//CPOX//TIMM9//TRIAP1//VDAC1//RBM7//PCGF3//USP16//POP4//KAT7//WDHD1//ZHX1//E2F5//DZIP1//NCBP2//KDM2A//PPP1R13B//CRTC1//PIP5K1C//GABPB1//MFSD8//SENP3//NR6A1//APEX1//KPNA1//AFF3//SMAD5//MITF//NFYC//NPM1//NMD3//DBR1//CTDSPL2//CDK12//PLAGL1//ATRX//QRICH1//COMMD8//UBE2W//MCM10//H2AFY2//RNPC3//PRKAA2//FAM222B//PSMA1//PSMC6//NUFIP2//WDR48//PTPN7//WIZ//RAD52//RARB//RNF4//RORA//NECAB1//HIF3A//COPS7B//C11ORF1//AEN//SNRPF//SP4//TAF11//TRPS1//UBE2I//XBP1//SAP30L//SLTM//ZC3H12A//DUSP16//CDK2AP1//PIP5K1A//MEX3B//STK24//CUL2//CDC14A//NCOA1//CDKL1//CCNC//PIAS2//FAM114A1//SRSF11//RASSF2//SUB1//SPC24//DDX6//LARP4B//RPL7L1//NF2//CMPK1//ARNTL2//REXO4//SCD//SLC30A5//UPF3A//TBCA//DDX3Y//EIF3A//MOB1B//MRPS7//GFI1//KLF2//MIXL1//HAND2//C14ORF39//SYCE1//PURB//DR1//FAS//TAF5L//APEX2//YBX2// |
| GO:0005730 | nucleolus | Cellular component | 31 | 931 | 414 | 18698 | 1.50385798865694 | 0.015810499271949 | 0.508420109039109 | 1.80105441548027 | 0.0748792270531401 | E2F5//APEX2//YBX2//CDK12//POP4//SUB1//SPC24//DDX6//LARP4B//SENP3//RPL7L1//APEX1//NF2//NPM1//NMD3//CMPK1//MCM10//ARNTL2//REXO4//RCN2//SCD//AEN//SLC30A5//UPF3A//TBCA//SAP30L//STK24//CUL2//DDX3Y//EIF3A//MOB1B// |
| GO:0030137 | COPI-coated vesicle | Cellular component | 3 | 25 | 414 | 18698 | 5.41971014492754 | 0.0172689370621702 | 0.508420109039109 | 1.76273439330926 | 0.0072463768115942 | TMEM199//COPG1//TMED7// |
| GO:0098797 | plasma membrane protein complex | Cellular component | 21 | 574 | 414 | 18698 | 1.65235065394132 | 0.0174574995666669 | 0.508420109039109 | 1.75801796006708 | 0.0507246376811594 | RGS7//CACNA2D4//GJB2//KCNC4//KCNQ3//KCNS1//STX1A//SNTG1//CDH24//CLTA//SYNJ1//FAS//FKBP1A//B2M//HLA-DPB1//CD3G//TFRC//CALCR//SHISA9//SHISA6//GRIK2// |
| GO:0044428 | nuclear part | Cellular component | 117 | 4435 | 414 | 18698 | 1.19148080976423 | 0.0175612196418381 | 0.508420109039109 | 1.75544532525644 | 0.282608695652174 | SNRPF//TMEM170A//AGPAT5//UBE2I//BCHE//KPNA1//RBM7//FSTL3//PCGF3//USP16//POP4//KAT7//WDHD1//ZHX1//E2F5//ALDH1B1//DZIP1//NCBP2//KDM2A//PPP1R13B//CRTC1//PIP5K1C//GABPB1//MFSD8//SENP3//NR6A1//APEX1//AFF3//SMAD5//MITF//NFYC//NPM1//OXCT1//NMD3//DBR1//CTDSPL2//TRIAP1//CDK12//PLAGL1//ATRX//QRICH1//COMMD8//UBE2W//MCM10//H2AFY2//RNPC3//PRKAA2//FAM222B//PSMA1//PSMC6//NUFIP2//WDR48//PTPN7//WIZ//RAD52//RARB//RNF4//RORA//NECAB1//HIF3A//COPS7B//C11ORF1//AEN//SP4//TAF11//TRPS1//XBP1//SAP30L//SLTM//ZC3H12A//DUSP16//CDK2AP1//PIP5K1A//MEX3B//STK24//CUL2//CDC14A//NCOA1//CDKL1//CCNC//PIAS2//FAM114A1//SRSF11//RASSF2//SUB1//SPC24//DDX6//LARP4B//RPL7L1//NF2//CMPK1//ARNTL2//REXO4//RCN2//SCD//SLC30A5//UPF3A//TBCA//DDX3Y//EIF3A//MOB1B//GFI1//OSBPL8//CERS6//STX1A//MAPKBP1//FAS//APEX2//YBX2//KLF2//MIXL1//HAND2//C14ORF39//SYCE1//PURB//DR1//TAF5L// |
| GO:0018995 | host | Cellular component | 5 | 69 | 414 | 18698 | 3.27277182664706 | 0.0183481063797583 | 0.508420109039109 | 1.736408750554 | 0.0120772946859903 | CLEC4M//CD209//KPNA1//DYNLT1//LMAN1// |
| GO:0043657 | host cell | Cellular component | 5 | 69 | 414 | 18698 | 3.27277182664706 | 0.0183481063797583 | 0.508420109039109 | 1.736408750554 | 0.0120772946859903 | LMAN1//CLEC4M//CD209//KPNA1//DYNLT1// |
| GO:0030135 | coated vesicle | Cellular component | 12 | 276 | 414 | 18698 | 1.96366309598824 | 0.0205241639871892 | 0.509622211092999 | 1.68773452400852 | 0.0289855072463768 | LMAN1//TMED7//CLTCL1//STEAP2//ATP7A//CLTA//TMEM199//HLA-DPB1//B2M//TFRC//CD3G//COPG1// |
| GO:0044444 | cytoplasmic part | Cellular component | 234 | 9626 | 414 | 18698 | 1.09790513012764 | 0.0212771547674129 | 0.509622211092999 | 1.67208644740269 | 0.565217391304348 | GTPBP1//CCSAP//TCTEX1D2//DNALI1//RIPK3//COX4I1//CPOX//CRAT//DDX6//DHFR//ALDH1B1//KIF1B//PPP1R13B//TIMM9//ACAD8//APEX2//APEX1//FAS//OXCT1//PCK1//TRIAP1//MARC2//RFK//AGPAT5//COA1//BAK1//MCCC2//HIF3A//DYNLT1//VDAC1//YWHAG//MUL1//NCOA4//SFXN1//EXOG//NAT2//ARPC5//NOD1//IFI30//KAT7//OSBPL8//CLTA//KLHDC1//MAP3K8//ASB15//PSMA8//SPC24//EXOC8//DGKA//DHCR24//DYNC1LI2//EIF1AX//EIF4E//EIF5//FKBP1A//COPG1//DZIP1//ALDOB//NCBP2//LARP4B//OTUD3//FBXW11//CRTC1//PIP5K1C//STEAP2//GJB2//GCLM//GSDMA//KRT6C//SATL1//INPP5B//KPNA1//AFF3//SULT6B1//SMAD5//MTM1//MYL1//NF2//NPM1//CRLF3//PDE1B//PEX5L//CMPK1//ATP7A//COMMD8//ETNK2//OGFOD1//PRKAA2//N4BP2//NGLY1//B2M//PSMA1//PSMC6//NUFIP2//PTPN7//RAP2C//ABCD3//ENOPH1//RGS7//RPE//NECAB1//COPS7B//UPF3A//SNRPF//SP4//STX1A//BTF3//PRDX2//TCHH//TSN//UBE2I//VSNL1//XPNPEP1//YES1//CSDE1//ARHGAP28//PTP4A2//DUSP16//CDK2AP1//CLTCL1//PIP5K1A//RAB6C//MEX3B//STK24//CUL2//KRTAP4-4//C1ORF198//RGS5//CDC14A//NCOA1//DDX3Y//EIF3A//SYNJ1//IDI2//MOB1B//FAM114A1//CEP135//RASSF2//G3BP2//WDR1//MRPS7//CPQ//TMEM199//CYP19A1//CERS6//SUMF2//C2ORF66//ASPH//TMED7//UFM1//TMEM100//RCN2//RPE65//SCD//NECAB3//THBS1//XBP1//CPED1//EBPL//CD1D//LMAN1//FSTL3//F2R//ZDHHC17//HEPACAM2//PLAGL1//FAM20C//SLC30A5//SMO//TAF11//GALNT14//LZTR1//RAB36//SNAP47//CHIC1//SERPINB3//TFRC//GABPB1//TAF5L//ZC3H12A//FAM168B//KCNS1//LRAT//TRAF4//MAP9//CCR5//SNX30//TRIM71//EXD1//LACC1//CUX2//HLA-DPB1//CAMK1G//KERA//SEC11A//LTBP1//BCHE//VWA1//COL23A1//H6PD//NUS1//TMEM170A//CANT1//LRRC8B//MOGAT3//AGPAT4//EMC4//TIMP2//RPL7L1//SPAG17//IGSF22//PYROXD1//CCDC68//CEP350//CTTNBP2//RNF152//WDR48//LAPTM5//CTSF//SAMD9L//SLC30A2//MFSD8//ACPP//SYP//C5AR1//CD3G//ATP6V0E1//VNN1//GZMK//NPPC//SLC25A35//GRIK2//ASZ1//COX18// |
| GO:0030286 | dynein complex | Cellular component | 4 | 49 | 414 | 18698 | 3.68687764961057 | 0.0229348103880408 | 0.509622211092999 | 1.63950484601676 | 0.00966183574879227 | DYNC1LI2//TCTEX1D2//DYNLT1//DNALI1// |
| GO:0034703 | cation channel complex | Cellular component | 10 | 217 | 414 | 18698 | 2.08130189897371 | 0.023132194254121 | 0.509622211092999 | 1.63578316933669 | 0.0241545893719807 | SHISA6//SHISA9//TRPC5//CACNA2D4//KCNC4//KCNQ3//KCNS1//STX1A//GRIK2//FKBP1A// |
| GO:0016442 | RISC complex | Cellular component | 2 | 11 | 414 | 18698 | 8.21168203776899 | 0.0235681531228163 | 0.509622211092999 | 1.62767444887135 | 0.00483091787439614 | DDX6//EIF4E// |
| GO:0031332 | RNAi effector complex | Cellular component | 2 | 11 | 414 | 18698 | 8.21168203776899 | 0.0235681531228163 | 0.509622211092999 | 1.62767444887135 | 0.00483091787439614 | DDX6//EIF4E// |
| GO:0044215 | other organism | Cellular component | 5 | 75 | 414 | 18698 | 3.0109500805153 | 0.0253368732682537 | 0.509622211092999 | 1.59624698085465 | 0.0120772946859903 | CLEC4M//CD209//KPNA1//DYNLT1//LMAN1// |
| GO:0044216 | other organism cell | Cellular component | 5 | 75 | 414 | 18698 | 3.0109500805153 | 0.0253368732682537 | 0.509622211092999 | 1.59624698085465 | 0.0120772946859903 | CLEC4M//CD209//KPNA1//DYNLT1//LMAN1// |
| GO:0044217 | other organism part | Cellular component | 5 | 75 | 414 | 18698 | 3.0109500805153 | 0.0253368732682537 | 0.509622211092999 | 1.59624698085465 | 0.0120772946859903 | CLEC4M//CD209//KPNA1//DYNLT1//LMAN1// |
| GO:0012506 | vesicle membrane | Cellular component | 26 | 781 | 414 | 18698 | 1.50354741536615 | 0.0258251753221221 | 0.509622211092999 | 1.58795672145706 | 0.0628019323671498 | KIF1B//ZDHHC17//YWHAG//HLA-DPB1//SLC30A5//C5AR1//SMO//LMAN1//B2M//SNAP47//CLTA//STX1A//ACPP//VNN1//RAP2C//TMEM199//TFRC//CD3G//INPP5B//ATP7A//ATP6V0E1//TMED7//CLTCL1//COPG1//SYP//SYNJ1// |
| GO:0043227 | membrane-bounded organelle | Cellular component | 298 | 12616 | 414 | 18698 | 1.06681569910458 | 0.0259041236145199 | 0.509622211092999 | 1.58663109613178 | 0.719806763285024 | CPQ//IFI30//ALDH1B1//ZDHHC17//MFSD8//APEX2//PLAGL1//WDR48//ABCD3//UPF3A//SMO//SLC30A2//CDKL1//SEC11A//RPE65//SERPINB3//RIPK3//COX4I1//CPOX//CRAT//DDX6//DHFR//KIF1B//PPP1R13B//TIMM9//ACAD8//APEX1//FAS//OXCT1//PCK1//TRIAP1//MARC2//RFK//AGPAT5//COA1//BAK1//MCCC2//HIF3A//DYNLT1//VDAC1//YWHAG//MUL1//NCOA4//SFXN1//EXOG//RBM7//FSTL3//SPEG//PCGF3//USP16//ZNF273//SUB1//BTG3//KAT7//ZHX1//FOXP4//ZNF488//ASB15//PSMA8//ZNF597//SPC24//ZNF558//IRX2//ZNF320//DHCR24//DR1//E2F5//FGF14//DZIP1//NCBP2//FBXW11//CUX2//CRTC1//EBF3//GABPB1//ALX3//SENP3//NR6A1//GFI1//TAF5L//ZNF544//HOXA9//TUBB2B//ZNF699//KPNA1//AFF3//SMAD5//MCAM//MITF//NF2//NFYC//NOVA2//NPM1//PAX7//PBX3//NMD3//YBX2//DBR1//TAOK3//CRLF3//UFM1//CMPK1//CDK12//ATP7A//SNTG1//ATRX//ACPP//OGFOD1//UBE2W//MCM10//RNPC3//PRKAA2//NGLY1//PSMA1//ARNTL2//PSMC6//REXO4//NUFIP2//PURB//ENOPH1//WIZ//RAD52//RARB//PBOV1//RFX4//RNF4//ALX4//RORA//ZNF704//NECAB3//NECAB1//C11ORF1//AEN//SLC30A5//SP4//TACC1//BTF3//TRPS1//TSN//UBE2I//XBP1//ZNF37A//ZNF75D//PGBD5//ZNF329//SAP30L//SLTM//PYROXD1//ZC3H12A//PTP4A2//DUSP16//CDK2AP1//MIXL1//PIP5K1A//RAB6C//C2ORF16//STK24//CDC14A//NCOA1//DDX3Y//EIF3A//CCNC//FUBP3//PIAS2//ZNF251//ZNF804A//MOB1B//FAM114A1//SRSF11//TEX30//QKI//CREB5//TRAF4//RASSF2//MFAP3L//CEP350//TMEM199//CYP19A1//COPG1//CERS6//SUMF2//C2ORF66//ASPH//TMED7//TMEM100//RCN2//SCD//THBS1//CPED1//EBPL//CD1D//GJB2//INPP5B//LMAN1//F2R//HEPACAM2//B2M//FAM20C//TAF11//YES1//CSDE1//GALNT14//LZTR1//RAB36//EDIL3//TFRC//CTSF//DYNC1LI2//RNF152//LAPTM5//LRAT//FKBP1A//LACC1//IDI2//CLTA//CHIC1//ARPC5//BTN2A2//SEMA3C//BTN2A1//CANT1//FAM168B//EIF4E//ALDOB//KRT6C//GPX3//CD274//CD209//MTM1//ARL15//H2AFY2//RAP2C//PTPRG//RPE//SERPINB4//VWA1//ST14//TBCA//PRDX2//TIMP2//XPNPEP1//XPNPEP2//CLTCL1//VNN1//WDR1//PEX5L//CCR5//STEAP2//SNX30//SAMD9L//EXOC8//STX1A//SYP//CTTNBP2//GZMK//NPPC//HLA-DPB1//KERA//C5AR1//SNAP47//CD3G//SYNJ1//ATP6V0E1//CAMK1G//SLC25A35//MRPS7//PIP5K1C//OSBPL8//TMEM170A//COX18//KLF2//HAND2//C14ORF39//SYCE1//SNRPF//BCHE//POP4//WDHD1//KDM2A//CTDSPL2//QRICH1//COMMD8//FAM222B//PTPN7//COPS7B//MEX3B//CUL2//MAPKBP1//LARP4B//RPL7L1//LTBP1//COL23A1//H6PD//NUS1//LRRC8B//MOGAT3//AGPAT4//EMC4// |
| GO:0044446 | intracellular organelle part | Cellular component | 221 | 9080 | 414 | 18698 | 1.0992620613335 | 0.0265242332377235 | 0.509622211092999 | 1.57635716182582 | 0.533816425120773 | FKBP1A//ZC3H12A//TMEM170A//AGPAT5//UBE2I//BCHE//CPOX//TIMM9//TRIAP1//DYNC1LI2//FBXO28//RASSF2//H2AFY2//SPC24//SNRPF//KPNA1//RBM7//FSTL3//PCGF3//USP16//POP4//KAT7//WDHD1//ZHX1//E2F5//ALDH1B1//DZIP1//NCBP2//KDM2A//PPP1R13B//CRTC1//PIP5K1C//GABPB1//MFSD8//SENP3//NR6A1//APEX1//AFF3//SMAD5//MITF//NFYC//NPM1//OXCT1//NMD3//DBR1//CTDSPL2//CDK12//PLAGL1//ATRX//QRICH1//COMMD8//UBE2W//MCM10//RNPC3//PRKAA2//FAM222B//PSMA1//PSMC6//NUFIP2//WDR48//PTPN7//WIZ//RAD52//RARB//RNF4//RORA//NECAB1//HIF3A//COPS7B//C11ORF1//AEN//SP4//TAF11//TRPS1//XBP1//SAP30L//SLTM//DUSP16//CDK2AP1//PIP5K1A//MEX3B//STK24//CUL2//CDC14A//NCOA1//CDKL1//CCNC//PIAS2//FAM114A1//SRSF11//SUB1//DDX6//LARP4B//RPL7L1//NF2//CMPK1//ARNTL2//REXO4//RCN2//SCD//SLC30A5//UPF3A//TBCA//DDX3Y//EIF3A//MOB1B//GFI1//OSBPL8//CERS6//STX1A//ACAD8//MCCC2//VDAC1//TCTEX1D2//WDR1//CCSAP//DNALI1//ALDOB//TACC1//YES1//CLTA//HEPACAM2//DYNLT1//CLTCL1//CEP350//IGSF22//KIF1B//ARPC5//MAP9//DHCR24//COPG1//CUX2//ZDHHC17//HLA-DPB1//LMAN1//TMED7//B2M//CAMK1G//GALNT14//RAB36//KERA//SEC11A//SUMF2//LTBP1//FAM20C//VWA1//THBS1//COL23A1//H6PD//NUS1//CANT1//TMEM199//CYP19A1//LRRC8B//MOGAT3//ASPH//AGPAT4//NECAB3//EBPL//CD1D//LRAT//EMC4//YWHAG//TIMP2//KLF2//MIXL1//HAND2//MAPKBP1//RNF152//ACPP//SLC30A2//LAPTM5//SERPINB3//IFI30//CTSF//PEX5L//ABCD3//CRAT//FBXW11//RAB6C//CEP135//COA1//MRPS7//MYL1//C5AR1//TFRC//CD3G//SMO//INPP5B//ATP7A//ATP6V0E1//VNN1//RAP2C//STEAP2//MARC2//BAK1//COX4I1//SLC25A35//CSDE1//SFXN1//EXOG//C14ORF39//SYCE1//PURB//APEX2//YBX2//FAS//KRT6C//CCDC68//SPAG17//TUBB2B//SYNJ1//SYP//TAF5L//KRTAP4-12//KRTAP4-4//ZNF804A//DR1//SNAP47//COX18//MUL1// |
| GO:0005777 | peroxisome | Cellular component | 7 | 131 | 414 | 18698 | 2.41335693476417 | 0.026697321884965 | 0.509622211092999 | 1.57353230226527 | 0.0169082125603865 | PEX5L//ABCD3//CRAT//LACC1//MARC2//MUL1//IDI2// |
| GO:0042579 | microbody | Cellular component | 7 | 131 | 414 | 18698 | 2.41335693476417 | 0.026697321884965 | 0.509622211092999 | 1.57353230226527 | 0.0169082125603865 | CRAT//LACC1//MARC2//ABCD3//MUL1//IDI2//PEX5L// |
| GO:0098796 | membrane protein complex | Cellular component | 36 | 1166 | 414 | 18698 | 1.39443657245134 | 0.0275087606833587 | 0.513696204934894 | 1.56052897487758 | 0.0869565217391304 | TRIAP1//SEC11A//BAK1//VDAC1//SYNJ1//SNAP47//STX1A//CLTA//CLTCL1//EMC4//ATP6V0E1//RGS7//CACNA2D4//GJB2//KCNC4//KCNQ3//KCNS1//SNTG1//CDH24//FAS//COX4I1//TIMM9//LRRC8B//TMEM199//FKBP1A//COPG1//TMED7//SHISA9//SHISA6//GRIK2//TRPC5//B2M//HLA-DPB1//CD3G//TFRC//CALCR// |
| GO:0044422 | organelle part | Cellular component | 226 | 9321 | 414 | 18698 | 1.09506713581664 | 0.0286255204538932 | 0.523177065316899 | 1.54324660847043 | 0.545893719806763 | SEC11A//RPE65//CDC14A//ACPP//SYNJ1//OSBPL8//CERS6//AEN//STX1A//RNF152//DDX6//CCSAP//TCTEX1D2//DNALI1//DZIP1//SMO//LYZL6//CCDC68//FKBP1A//IGSF22//MYL1//LMAN1//PYROXD1//MTM1//GRIK2//HLA-DPB1//SLC30A5//C5AR1//KIF1B//ZDHHC17//YWHAG//COX4I1//CPOX//CRAT//TIMM9//SLC25A35//MRPS7//MARC2//ABCD3//VDAC1//CSDE1//SFXN1//EXOG//DHCR24//COPG1//CUX2//HEPACAM2//TMED7//B2M//CAMK1G//GALNT14//RAB36//AGPAT5//BAK1//PIP5K1C//STEAP2//TFRC//ATP6V0E1//CD1D//ZC3H12A//ASPH//TMEM199//SYP//COX18//COA1//MUL1//TMEM170A//UBE2I//BCHE//TRIAP1//ARPC5//DYNLT1//ATP7A//CLTCL1//ALDH1B1//ACAD8//OXCT1//MCCC2//FSTL3//SUMF2//LTBP1//FAM20C//RCN2//VWA1//THBS1//COL23A1//H6PD//KERA//CANT1//TIMP2//RPL7L1//WDHD1//DYNC1LI2//FBXO28//RASSF2//SPC24//ATRX//APEX1//H2AFY2//KLF2//TRPS1//MIXL1//NCOA1//HAND2//C14ORF39//SYCE1//PURB//MCM10//SAP30L//KAT7//E2F5//APEX2//YBX2//CDK12//SNRPF//KPNA1//RBM7//PCGF3//USP16//POP4//ZHX1//NCBP2//KDM2A//PPP1R13B//CRTC1//GABPB1//MFSD8//SENP3//NR6A1//AFF3//SMAD5//MITF//NFYC//NPM1//NMD3//DBR1//CTDSPL2//PLAGL1//QRICH1//COMMD8//UBE2W//RNPC3//PRKAA2//FAM222B//PSMA1//PSMC6//NUFIP2//WDR48//PTPN7//WIZ//RAD52//RARB//RNF4//RORA//NECAB1//HIF3A//COPS7B//C11ORF1//SP4//TAF11//XBP1//SLTM//DUSP16//CDK2AP1//PIP5K1A//MEX3B//STK24//CUL2//CDKL1//CCNC//PIAS2//FAM114A1//SRSF11//SUB1//LARP4B//NF2//CMPK1//ARNTL2//REXO4//SCD//UPF3A//TBCA//DDX3Y//EIF3A//MOB1B//GFI1//FAS//MAPKBP1//KRT6C//WDR1//FBXW11//RAB6C//CEP135//CEP350//ALDOB//TACC1//YES1//CLTA//SPAG17//TUBB2B//MAP9//NUS1//CYP19A1//LRRC8B//MOGAT3//AGPAT4//NECAB3//EBPL//LRAT//EMC4//SERPINB3//IFI30//CTSF//PEX5L//CD3G//INPP5B//VNN1//RAP2C//DR1//TAF5L//KRTAP4-12//KRTAP4-4//SLC30A2//LAPTM5//SNAP47//ZNF804A// |
| GO:0005634 | nucleus | Cellular component | 180 | 7267 | 414 | 18698 | 1.11869619064143 | 0.0295102376335198 | 0.528110294316531 | 1.53002729329534 | 0.434782608695652 | SAP30L//KAT7//ATRX//APEX1//H2AFY2//KLF2//TRPS1//MIXL1//NCOA1//HAND2//UBE2I//C14ORF39//SYCE1//E2F5//APEX2//YBX2//CDK12//SNRPF//TMEM170A//AGPAT5//BCHE//KPNA1//RBM7//FSTL3//PCGF3//USP16//POP4//WDHD1//ZHX1//ALDH1B1//DZIP1//NCBP2//KDM2A//PPP1R13B//CRTC1//PIP5K1C//GABPB1//MFSD8//SENP3//NR6A1//AFF3//SMAD5//MITF//NFYC//NPM1//OXCT1//NMD3//DBR1//CTDSPL2//TRIAP1//PLAGL1//QRICH1//COMMD8//UBE2W//MCM10//RNPC3//PRKAA2//FAM222B//PSMA1//PSMC6//NUFIP2//WDR48//PTPN7//WIZ//RAD52//RARB//RNF4//RORA//NECAB1//HIF3A//COPS7B//C11ORF1//AEN//SP4//TAF11//XBP1//SLTM//ZC3H12A//DUSP16//CDK2AP1//PIP5K1A//MEX3B//STK24//CUL2//CDC14A//CDKL1//CCNC//PIAS2//FAM114A1//SRSF11//RASSF2//PURB//DR1//MAPKBP1//SUB1//SPC24//DDX6//LARP4B//RPL7L1//NF2//CMPK1//ARNTL2//REXO4//RCN2//SCD//SLC30A5//UPF3A//TBCA//DDX3Y//EIF3A//MOB1B//GFI1//FAS//TAF5L//OSBPL8//CERS6//STX1A//SPEG//ZNF273//BTG3//FOXP4//ZNF488//COX4I1//ASB15//PSMA8//ZNF597//ZNF558//IRX2//ZNF320//DHCR24//FGF14//FBXW11//CUX2//EBF3//ALX3//ZNF544//HOXA9//TUBB2B//ZNF699//MCAM//NOVA2//PAX7//PBX3//TAOK3//CRLF3//UFM1//ATP7A//SNTG1//ACPP//OGFOD1//NGLY1//ENOPH1//PBOV1//RFX4//ALX4//ZNF704//SERPINB3//NECAB3//TACC1//BTF3//TSN//VDAC1//ZNF37A//ZNF75D//PGBD5//ZNF329//PYROXD1//NCOA4//PTP4A2//RAB6C//C2ORF16//FUBP3//ZNF251//ZNF804A//TEX30//QKI//CREB5//TRAF4//MFAP3L//CEP350// |
| GO:0030424 | axon | Cellular component | 19 | 538 | 414 | 18698 | 1.5950200240648 | 0.0314842117436813 | 0.539147168472007 | 1.50190717546514 | 0.0458937198067633 | ARPC5//EXOC8//TIMP2//TRPC5//ZNF804A//KCNQ3//GRIK2//SYP//SYNJ1//FKBP1A//KCNC4//DYNLT1//KIF1B//CCSAP//FAM168B//HEPACAM//CADM2//STX1A//MUL1// |
| GO:0030130 | clathrin coat of trans-Golgi network vesicle | Cellular component | 2 | 13 | 414 | 18698 | 6.94834633965069 | 0.0324660588708865 | 0.539147168472007 | 1.48857042817444 | 0.00483091787439614 | CLTA//CLTCL1// |
| GO:0032591 | dendritic spine membrane | Cellular component | 2 | 13 | 414 | 18698 | 6.94834633965069 | 0.0324660588708865 | 0.539147168472007 | 1.48857042817444 | 0.00483091787439614 | SHISA6//SHISA9// |
| GO:0030659 | cytoplasmic vesicle membrane | Cellular component | 25 | 761 | 414 | 18698 | 1.48371390301345 | 0.0326375468690854 | 0.539147168472007 | 1.48628249150081 | 0.0603864734299517 | HLA-DPB1//SLC30A5//C5AR1//SMO//LMAN1//B2M//ACPP//VNN1//RAP2C//TMEM199//TFRC//CD3G//INPP5B//ATP7A//ATP6V0E1//SNAP47//CLTA//STX1A//TMED7//CLTCL1//COPG1//SYP//KIF1B//ZDHHC17//YWHAG// |
| GO:0030120 | vesicle coat | Cellular component | 4 | 55 | 414 | 18698 | 3.2846728151076 | 0.0333286426765158 | 0.540175548285416 | 1.47718237281119 | 0.00966183574879227 | COPG1//TMED7//CLTA//CLTCL1// |
| GO:0043231 | intracellular membrane-bounded organelle | Cellular component | 259 | 10864 | 414 | 18698 | 1.07672506100901 | 0.0347198298855669 | 0.552302479105592 | 1.45942241137402 | 0.6256038647343 | RIPK3//COX4I1//CPOX//CRAT//DDX6//DHFR//ALDH1B1//KIF1B//PPP1R13B//TIMM9//ACAD8//APEX2//APEX1//FAS//OXCT1//PCK1//TRIAP1//MARC2//RFK//AGPAT5//COA1//BAK1//MCCC2//HIF3A//DYNLT1//VDAC1//YWHAG//MUL1//NCOA4//SFXN1//EXOG//RBM7//FSTL3//SPEG//PCGF3//USP16//ZNF273//SUB1//BTG3//KAT7//ZHX1//FOXP4//ZNF488//ASB15//PSMA8//ZNF597//SPC24//ZNF558//IRX2//ZNF320//DHCR24//DR1//E2F5//FGF14//DZIP1//NCBP2//FBXW11//CUX2//CRTC1//EBF3//GABPB1//ALX3//SENP3//NR6A1//GFI1//TAF5L//ZNF544//HOXA9//TUBB2B//ZNF699//KPNA1//AFF3//SMAD5//MCAM//MITF//NF2//NFYC//NOVA2//NPM1//PAX7//PBX3//NMD3//YBX2//DBR1//TAOK3//CRLF3//UFM1//CMPK1//CDK12//PLAGL1//ATP7A//SNTG1//ATRX//ACPP//OGFOD1//UBE2W//MCM10//RNPC3//PRKAA2//NGLY1//PSMA1//ARNTL2//PSMC6//REXO4//NUFIP2//WDR48//PURB//ENOPH1//WIZ//RAD52//RARB//PBOV1//RFX4//RNF4//ALX4//RORA//ZNF704//SERPINB3//NECAB3//NECAB1//C11ORF1//AEN//SLC30A5//UPF3A//SP4//TACC1//BTF3//TRPS1//TSN//UBE2I//XBP1//ZNF37A//ZNF75D//PGBD5//ZNF329//SAP30L//SLTM//PYROXD1//ZC3H12A//PTP4A2//DUSP16//CDK2AP1//MIXL1//PIP5K1A//RAB6C//C2ORF16//STK24//CDC14A//NCOA1//DDX3Y//EIF3A//CDKL1//CCNC//FUBP3//PIAS2//ZNF251//ZNF804A//MOB1B//FAM114A1//SRSF11//TEX30//QKI//CREB5//TRAF4//RASSF2//MFAP3L//CEP350//CPQ//TMEM199//CYP19A1//COPG1//CERS6//SUMF2//C2ORF66//ASPH//TMED7//TMEM100//RCN2//RPE65//SCD//THBS1//CPED1//EBPL//CD1D//GJB2//INPP5B//LMAN1//F2R//ZDHHC17//HEPACAM2//B2M//FAM20C//SMO//TAF11//YES1//CSDE1//GALNT14//LZTR1//RAB36//LRAT//FKBP1A//LACC1//ABCD3//IDI2//IFI30//DYNC1LI2//RNF152//LAPTM5//CTSF//PEX5L//MFSD8//HLA-DPB1//SLC30A2//KERA//CLTA//VNN1//SLC25A35//MRPS7//COX18//H2AFY2//KLF2//HAND2//C14ORF39//SYCE1//SNRPF//TMEM170A//BCHE//POP4//WDHD1//KDM2A//PIP5K1C//CTDSPL2//QRICH1//COMMD8//FAM222B//PTPN7//COPS7B//MEX3B//CUL2//MAPKBP1//LARP4B//RPL7L1//TBCA//OSBPL8//STX1A//SEC11A//LTBP1//VWA1//COL23A1//H6PD//NUS1//CANT1//LRRC8B//MOGAT3//AGPAT4//EMC4//CAMK1G//CLTCL1//STEAP2// |
| GO:0150034 | distal axon | Cellular component | 11 | 268 | 414 | 18698 | 1.85375657942173 | 0.037038361328556 | 0.56292630145362 | 1.43134823585912 | 0.0265700483091787 | ARPC5//EXOC8//TIMP2//TRPC5//ZNF804A//FKBP1A//KCNC4//DYNLT1//GRIK2//SYP//SYNJ1// |
| GO:0005845 | mRNA cap binding complex | Cellular component | 2 | 14 | 414 | 18698 | 6.4520358868185 | 0.0373319485633955 | 0.56292630145362 | 1.42791934122171 | 0.00483091787439614 | EIF4E//NCBP2// |
| GO:0030658 | transport vesicle membrane | Cellular component | 9 | 203 | 414 | 18698 | 2.00235596487471 | 0.0373536661034416 | 0.56292630145362 | 1.42766676760066 | 0.0217391304347826 | HLA-DPB1//LMAN1//B2M//SNAP47//CLTA//STX1A//TMED7//CLTCL1//SYP// |
| GO:0000932 | P-body | Cellular component | 5 | 84 | 414 | 18698 | 2.68834828617437 | 0.0386733832221717 | 0.57276614116975 | 1.41258783334589 | 0.0120772946859903 | TRIM71//DDX6//EIF4E//ZC3H12A//MEX3B// |
| GO:0044432 | endoplasmic reticulum part | Cellular component | 39 | 1319 | 414 | 18698 | 1.33541000538396 | 0.0398042402773998 | 0.579522752513329 | 1.40007066081612 | 0.0942028985507246 | TRIAP1//SEC11A//FSTL3//SUMF2//LTBP1//B2M//FAM20C//BCHE//RCN2//VWA1//THBS1//COL23A1//H6PD//OSBPL8//NUS1//TMEM170A//CANT1//TMEM199//CYP19A1//DHCR24//COPG1//LRRC8B//CERS6//MOGAT3//LMAN1//ASPH//TMED7//AGPAT5//AGPAT4//SCD//NECAB3//XBP1//EBPL//CD1D//LRAT//ZC3H12A//EMC4//FKBP1A//HLA-DPB1// |
| GO:0012510 | trans-Golgi network transport vesicle membrane | Cellular component | 2 | 15 | 414 | 18698 | 6.0219001610306 | 0.0424565863055252 | 0.597872256335183 | 1.37205492793435 | 0.00483091787439614 | CLTA//CLTCL1// |
| GO:0030126 | COPI vesicle coat | Cellular component | 2 | 15 | 414 | 18698 | 6.0219001610306 | 0.0424565863055252 | 0.597872256335183 | 1.37205492793435 | 0.00483091787439614 | COPG1//TMED7// |
| GO:0030133 | transport vesicle | Cellular component | 14 | 381 | 414 | 18698 | 1.65957878453599 | 0.0452316473758018 | 0.626677178964738 | 1.34455759458458 | 0.0338164251207729 | HLA-DPB1//STEAP2//ATP7A//STX1A//SYP//VDAC1//CTTNBP2//SNAP47//CLTA//LMAN1//B2M//TMED7//CLTCL1//COPG1// |
| GO:0034518 | RNA cap binding complex | Cellular component | 2 | 16 | 414 | 18698 | 5.64553140096618 | 0.0478261883226254 | 0.64982910254347 | 1.32033423038488 | 0.00483091787439614 | EIF4E//NCBP2// |
| GO:0012507 | ER to Golgi transport vesicle membrane | Cellular component | 4 | 62 | 414 | 18698 | 2.91382265856319 | 0.0484156723664518 | 0.64982910254347 | 1.31501403255982 | 0.00966183574879227 | TMED7//HLA-DPB1//LMAN1//B2M// |
